# Pricing timer options and variance derivatives with closed-form partial transform under the 3/2 model

**DOI:** 10.1080/1350486X.2017.1285242

**Published:** 2017-02-06

**Authors:** Wendong Zheng, Pingping Zeng

**Affiliations:** ^a^ Department of Mathematics, Hong Kong University of Science and Technology, Hong Kong, China; ^b^ Department of Mathematics, Southern University of Science and Technology, Shenzhen, China

**Keywords:** 3/2 model, triple joint transition density, timer options, variance derivatives, discrete monitoring

## Abstract

Most of the empirical studies on stochastic volatility dynamics favour the 3/2 specification over the square-root (CIR) process in the Heston model. In the context of option pricing, the 3/2 stochastic volatility model (SVM) is reported to be able to capture the volatility skew evolution better than the Heston model. In this article, we make a thorough investigation on the analytic tractability of the 3/2 SVM by proposing a closed-form formula for the partial transform of the triple joint transition density 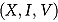 which stand for the log asset price, the quadratic variation (continuous realized variance) and the instantaneous variance, respectively. Two distinct formulations are provided for deriving the main result. The closed-form partial transform enables us to deduce a variety of marginal partial transforms and characteristic functions and plays a crucial role in pricing discretely sampled variance derivatives and exotic options that depend on both the asset price and quadratic variation. Various applications and numerical examples on pricing moment swaps and timer options with discrete monitoring feature are given to demonstrate the versatility of the partial transform under the 3/2 model.

## 1. Introduction

Stochastic volatility models (SVMs) were introduced to option pricing theory to resolve the incapability of the Black–Scholes framework in capturing the volatility smile/skew. Despite the difference in the specific assumption on the instantaneous volatility dynamics, the common practice of randomizing the volatility is to model the instantaneous volatility/variance as a correlated diffusion process. As pointed out by Itkin (2013), classic SVMs use a constant elasticity of variance (CEV) process for the instantaneous variance and there are just a few choices for the CEV parameter 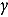 that exhibit mathematical tractability. Nevertheless, various versions of SVMs have been proposed in the literature. Hull and White (1987) model the instantaneous variance process as a geometric Brownian motion (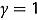). Scott (1987) and Chesney and Scott (1989) let the log variance process be a mean-reverting Ornstein–Uhlenbeck (OU) process. Stein and Stein (1991) and Schöbel and Zhu (1999) assume that the instantaneous volatility follows a mean-reverting OU process (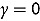). Heston (1993) instead proposes a mean-reverting square root process (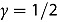) for the instantaneous variance. Barndorff-Nielsen and Shephard (2001) use a mean-reverting OU process to model the instantaneous variance process. Other variations of SVMs can be found in Bates (1996), Carr et al. (2003), Carr and Wu (2003), etc. Among all the proposed SVMs, the Heston model and its variants are the most popular ones. It is known that the Heston-type SVMs are essentially a subclass of the affine model family (see Duffie, Pan and Singleton (2000)).

Despite its popularity, there is a surprisingly large amount of empirical studies that report inconsistency of the affine models with market observations. Poteshman (1998) studies S&P500 index option prices over a 7-year period and concludes that both the physical and risk-neutral drifts of the instantaneous variance are nonaffine. Also, the volatility of variance is observed to be an increasing convex function of the instantaneous variance. Using an affine drift CEV process for the instantaneous variance to fit the S&P500 daily returns over a 30-year period, Ishida and Engle (2002) estimate the CEV parameter 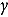 to be 1.71. In a similar work by Jones (2003), 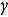 is found to be 1.33 for daily S&P100 returns and implied volatilities over a 14-year period. Chacko and Viceira (2003) employ the technique of the generalized method of moments on a 35-year period of weekly returns and a 71-year period of monthly returns and estimate the CEV power to be 1.10 and 1.65, respectively. Javaheri (2004) tests three CEV power values: 
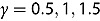
 on the time series of S&500 daily returns and finds that 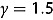 outperforms the other two. Using the same data as in Jones (2003), Bakshi, Ju and Ou-Yang (2006) test several SVMs on the time series of S&P100 implied volatilities and find that a linear drift model is rejected and the coefficient of the quadratic term is highly significant. Moreover, their estimate of the CEV power is 1.27. The implication of these empirical findings is that one should use a diffusion process with nonaffine drift and CEV power to be greater than 1. With a quadratic drift and CEV power equal to 1.5, the 3/2 SVM is obviously more firmly supported by the empirical study than the affine models.

Interestingly, the 3/2 model is not a brand-new model. In fact, it has already been examined by Heston (1997) and Lewis (2000) for its analytic tractability, applied to construct short rate models by Ahn and Gao (1999) and used to model credit default intensity by Andreasen (2001). However, until very recently the 3/2 model has not been considered as a mainstream SVM. The growing attention from the academia to the 3/2 model is partially attributed to the increasing interest in consistently modelling equity and volatility markets (represented by VIX), as a result of the booming of the volatility derivative market. In a recent work by Carr and Sun (2007), a new framework for option pricing that directly models the variance swap rate is proposed. They argue that the 3/2 specification for the instantaneous variance is a direct consequence of the model consistency requirement. Following Carr and Sun’s work, Itkin and Carr (2010) introduce the 3/2 power time change process, and Chan and Platen (2015) price long-dated variance swaps under the 3/2 SVM. By performing extensive numerical comparisons between the Heston model and the 3/2 model, Drimus (2012) reports that the 3/2 model is superior to the Heston model in the sense that it is able to predict upward-sloping volatility of variance smiles, which is in good consistency with market observations. Most recently, Goard and Mazur (2013) report strong empirical evidence that Volatility Index (VIX) follows a 3/2 process other than an affine square root process. In an effort to consistently modelling VIX and equity derivatives, Baldeaux and Badran (2014) consider a 3/2 plus jumps model for pricing VIX derivatives. Yuen, Zheng and Kwok (2015) derive closed-form pricing formulas for exotic variance swaps under the 3/2 model.

Analytic tractability of an underlying model is essential for derivatives pricing. As for the 3/2 model, the characteristic function of the log asset price process is derived in Heston (1997) and Lewis (2000). In order to facilitate the pricing of volatility derivatives, Carr and Sun (2007) obtain the joint characteristic function of the log asset price and the quadratic variation, and Baldeaux and Badran (2014) extend their result to the 3/2 plus jumps model. Lewis (2009) derives the joint transition density of the log asset price and the instantaneous variance for the 3/2 model with constant parameters. So far as we are informed, the full characterization of the joint distribution of the triple 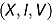, which stand for the log asset price, quadratic variation and the instantaneous variance, has not yet been done in the literature. Our paper fills this gap by providing a complete description of the joint distribution through the closed-form partial transform of the triple transition density. Two distinct formulations are proposed for deriving our main result. Inspired by the work by Carr and Sun (2007) and Lewis (2009), our partial differential equation (PDE) method follows a different approach from theirs. More specifically, Carr and Sun (2007) solve the governing PDE with an ingenious choice of substituting variable and then reduce the PDE to an ordinary differential equation (ODE) and Lewis (2009) uses a sequence of transformations to reduce the original governing PDE to a first-order linear PDE which is solvable by the standard method of characteristics. Motivated by the joint exponential affine structure of the log asset price and quadratic variation, we define the partial transform of the triple joint density function and then solve the governing PDE by converting it to a Riccati system of ordinary differential equations through a Laplace transform. In the probabilistic formulation, the partial transform is factorized as a product of the conditional characteristic function of the integrated variance and the marginal transition density of the instantaneous variance. We then find the explicit expressions for both terms by using the change of measure technique and the reciprocal relation between the 3/2 process and CIR process.

Put in the relatively thin collection of literature on option pricing under the 3/2 model, the contribution of our work is threefold. First, we obtain a closed-form formula of the partial transform of the triple transition density under the 3/2 model with a time-dependent mean reversion parameter. The closed-form partial transform fully characterizes the joint distribution of the triple 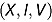 under the 3/2 model, and most of the existing analytic formulas for the joint density functions and characteristic functions in the literature can be viewed as marginal versions of the newly derived partial transform. Second, we describe a general transform-based analytic pricing methodology that relies on the newly derived closed-form partial transform and that works for a variety of exotic derivative products whose terminal payoffs have dependency on the asset price and quadratic variation. Specifically, we demonstrate how the finite-maturity discrete timer options and discretely sampled weighted moment swaps can be priced in closed-form formulas without any analytic or numerical approximations. Last but not least, we provide two distinct derivations of the closed-form formula of the partial transform. Our PDE approach takes advantage of the affine property of the pair 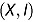 and explicitly solves the governing PDE by converting the equation into a Riccati system of ODEs. Our probabilistic approach explores the relationship between the partial transform and the conditional characteristic function of the integrated variance and works out the closed-form expression for the latter with the change of measure technique.

The rest of the paper is structured as follows. In Section 2, we provide a detailed description of the 3/2 model and its financial intuition. Section 3 is devoted to the derivation of our main result on the partial transform of the triple joint transition density under the PDE and probabilistic formulations. We also show that most univariate and bivariate characteristic and density functions under the 3/2 model can be derived immediately from the partial transform of the triple. Section 4 covers examples for demonstrating the applications in derivatives pricing under the 3/2 model. In particular, the pricing of finite-maturity discrete timer options and discretely sampled weighted moment swaps are investigated in details. Numerical experiments and analyses are provided in Section 5 and conclusive remarks are given in Section 6.

## 2. The 3/2 model

Consider the 3/2 SVM specified as follows:

(2.1)
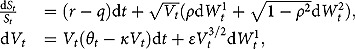

where 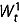 and 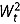 are two independent Brownian motions under the pricing measure *Q*. Here, we assume a constant risk-free rate *r* and dividend yield *q*. Time-dependent deterministic risk-free rate and dividend yield can be accommodated without difficulty. In contrast to the square root process with affine drift in the Heston model, the parameters in the 3/2 variance dynamics need to be interpreted differently. The speed of mean reversion is now 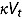, which is linear in 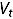 and is a stochastic quantity. Since 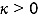 under usual scenarios, the mean correction is quicker when the instantaneous variance is higher. Also, 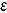 cannot be interpreted as the same volatility of variance as in the Heston model. In fact, one needs to multiply it by a scaling factor 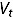 in order to make it comparable to its counterpart in the Heston model. The long-term mean reversion level is given by 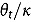. As pointed out by Itkin and Carr (2010), 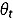 could be an independent stochastic process in the most general setting. By conditioning on the path of 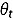, the analytic tractability of the 3/2 model with stochastic mean reversion level remains intact. Although in practice 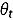 is often taken to be constant, this flexibility allows for more delicate modelling of the instantaneous variance dynamics when necessary. Thus, in this paper, we assume 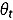 to be a deterministic function of time, but an extension to an independent random process 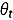 is not difficult.

Similar to the Heston model, not all choices of model parameters are admissible, in the sense that the non-explosion of 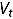 and the martingale property of the discounted asset price process are guaranteed. According to Drimus (2012), the parameters of the 3/2 model specified by (1) are constrained by

(2.2)
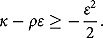

Notice that under normal market condition, 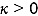 and 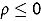, the above inequality is automatically satisfied.

## 3. The closed-form partial transform

In this section, we present our main result on the closed-form formula for the partial transform defined previously. On top of deriving the main result by solving the governing PDE analytically, we provide an insightful probabilistic argument and some interesting intermediate results are obtained as a by-product. We also briefly discuss how to deduce various marginal characteristic functions and transforms from the main result.

### 3.1. The main result

Let 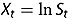 be the log asset price and 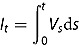 be the quadratic variation of the log asset price process. Let 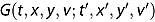 be the joint transition density of the triple 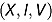 from state 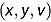 at time *t* to state 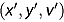 at a subsequent time 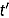. By the Feynman–Kac theorem, *G* satisfies the following Kolmogorov backward equation:

(3.1)
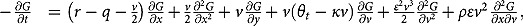

subject to the terminal condition:

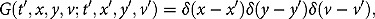

where 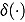 is the Dirac delta function.

The *partial transform* of *G*, denoted by 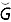, is defined by

(3.2)
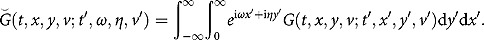

The reason for introducing 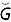 is that the affine structure of the pair 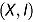 makes 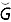 more tractable than *G* itself. Also, 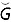 is more convenient to be used in option pricing. Obviously, 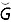 also satisfies (3.1) with the terminal condition being 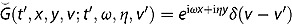. Since (3.1) has no coefficient involving *x* or *y*, it is natural to come up with the following solution form:

(3.3)
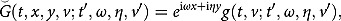

where *g* satisfies the following PDE:

(3.4)
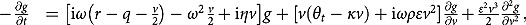

subject to the terminal condition:

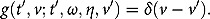

From (3.4), we see that *g* is essentially a function of *v* and *t*, while all the other variables affect *g* either through the PDE coefficients or boundary condition.

**Theorem 1**. *Under the 3/2 stochastic volatility model specified by (2.1), the partial transform of the triple joint transition density function defined by (3.4) is found to be*

(3.5)
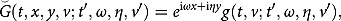

where

(3.6)
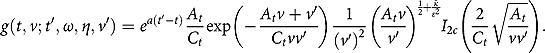

Here,

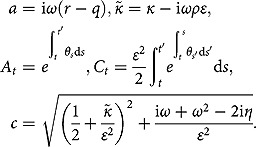

*Proof*. We prove it by explicitly solving (3.4). The details are given in Appendix A.

Remark 1. *The solution form of* 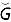 *given by (3.3) holds for any stochastic volatility process. In fact, if we consider a general stochastic volatility model specified by*

(3.7)
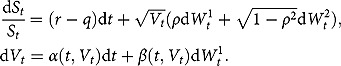

It can be shown in a similar manner that the governing equation for *g* is given by

(3.8)
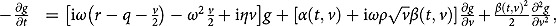

subject to the terminal condition: 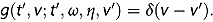 The partial transform of the model (3.7) admits a closed-form expression if (3.8) can be solved explicitly.

Remark 2. *Theorem 1 can be easily extended to incorporate jumps in the asset price process. The analytic formula under the 3/2 plus jumps model is given in* Appendix B.

#### 3.1.1. A probabilistic formulation of the partial transform

The partial transform defined by (3.2) admits an interesting conditional factorization, from which we can derive our main result in a pure probabilistic manner. Let 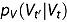 be the transition density function of the instantaneous variance for any 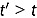 under the pricing measure *Q*. Since 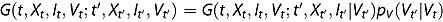, we have

(3.9)
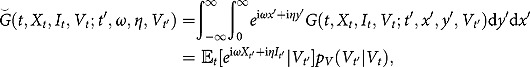

where 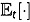 is short for 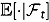 under the measure *Q* with 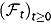 being the natural filtration. The dynamics in (1) can be rewritten to give (e.g., Zeng, Kwok and Zheng (2015))

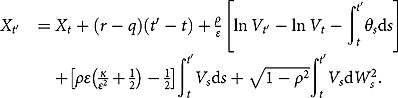

With this expression, the conditional expectation can be reformulated as

(3.10)
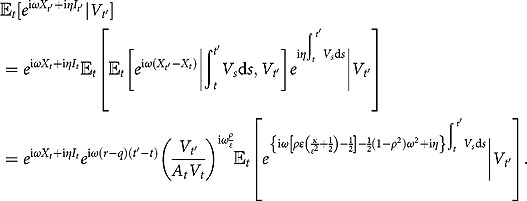

Therefore, the original problem boils down to the computation of the transition density of the variance process 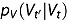 and the conditional characteristic function of the integrated variance in the form of 
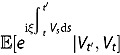
.

##### 3.1.1.1. Transition density of the instantaneous variance

It is well known that the reciprocal of the 3/2 process is a CIR process. Write 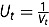, then 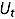 is given by the following inhomogeneous CIR process:

(3.11)
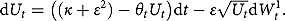

**Lemma 1**. *For any* 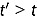, 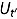 *follows a non-central chi-square distribution conditional on* 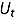. *Its transition (conditional) density function is given by*

(3.12)
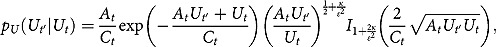

*where* 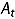 *and* 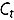 *are given in Theorem 1.*

*Proof*. The inhomogeneous CIR process (3.11) equals a squared Bessel process 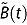 transformed by the following space-time changes in distribution

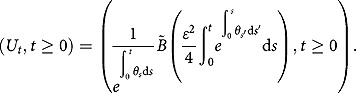

Based on the above relation and transition density of the squared Bessel process, Jeanblanc, Yor and Chesney (2009) provide the transition density of the inhomogeneous CIR process. We refer interested readers to Jeanblanc, Yor and Chesney (2009) for more details.

It follows that the transition density of 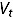 can be inferred to be

(3.13)
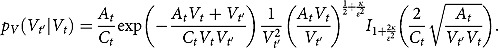

##### 3.1.1.2. Conditional characteristic function of the integrated variance

Because of the strong path dependency of 
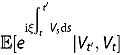
, the evaluation of this conditional expectation poses a major challenge. Fortunately, with the change of probability measure technique, we manage to find the analytic formula for this conditional expectation.

**Theorem 2** *Under the 3/2 stochastic volatility model specified by (1), the characteristic function of the integrated variance conditional on* 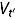 *admits*

(3.14)
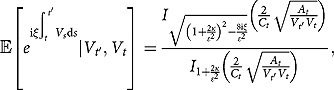

where the notations 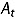 and 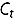 are the same as those given in Theorem 1.

*Proof*. The proof relies on a smart choice of a new probability measure under which the evaluation of expectations can be done conveniently. The details can be found in Appendix C.

Remark 3. *Formula (3.14) can be viewed as a generalization of* Baldeaux (2012) *for a time-dependent mean reversion parameter* 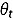. *Baldeaux’s derivation is based on Lie symmetry and Laplace transform method, whereas in this article we manage to propose an easier alternative to obtain a more general result. With (3.14), one can now construct the exact simulation of the asset price process under the 3/2 model with a time-dependent mean reversion parameter.*

One can now easily verify that combining (3.9), (3.10), (3.13) and (3.14) immediately leads to (3.5).

### 3.2. Marginal characteristic functions and transforms

Since the newly obtained partial transform characterizes the joint distribution of the triple, the existing univariate densities or bivariate densities as well as their Fourier transforms can be derived immediately from Theorem 1.

**Corollary 1** (Carr and Sun 2007) *Under the 3/2 sochastic volatility model specified by (2.1), the joint characteristic function of the pair* 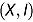 *conditional on* 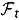 *is given by*

(3.15)
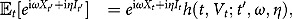

*where*

(3.16)
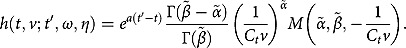

*Here*,

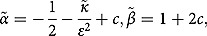

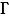
 is the gamma function, and *M* is the confluent hypergeometric function of the first kind.

*Proof*. See Appendix D.

Remark 4. *The marginal characteristic functions of X and I can be deduced by simply setting* 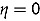 *and* 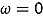, *respectively.*

Denote the joint transition density function of 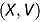 by 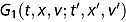 and define its partial transform by

(3.17)
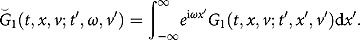

**Corollary 2**. *Under the 3/2 stochastic volatility model specified by (1), the partial transform defined by (19) is given by*

(3.18)
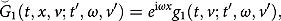

where

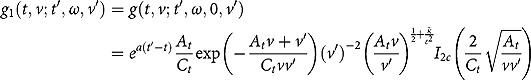

with

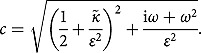

*Proof*. By virtue of (3.2), 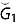 can be obtained by simply setting 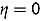 in (3.5).

Remark 5. *One can immediately see that this result can be viewed as a generalization of* Lewis (2009) *for a time-dependent mean reversion parameter* 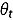.

Remark 6. *By further setting* 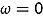 *in (3.18), we can retrieve the transition density of* 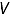:

(3.19)
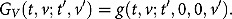

*This provides an alternative derivation of (3.13).*

## 4. Applications in derivatives pricing

In this section, we demonstrate how the partial transform of the triple and its induced marginal characteristic functions and transforms can be used to price various exotic derivative products that may be embedded with path-dependent features, and have a complicated terminal payoff structure that depends on the asset price and quadratic variation. The basic idea is that the risk-neutral expectations involved in the pricing of such products can always be calculated by integrating in the real domain against the density function, or alternatively, in the Fourier domain against the characteristic function. Once we have the closed-form formulas of the density or characteristic functions, the integrations can be carried out in a rather accurate and efficient manner.

### 4.1. Finite-maturity discrete timer options

Instead of having a deterministic expiry date as in a vanilla option, a discrete timer option with finite maturity expires on a random date which is either the first time when a pre-specified variance budget is fully consumed by the realized variance of the underlying asset price or the pre-specified mandatory expiry date, depending on which one comes earlier. Let *T* be the mandatory expiry of the timer option and denote the tenor of the monitoring dates for the realized variance by 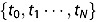. For brevity, we assume equally spaced monitoring interval, i.e., 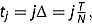 for 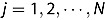.

At the initiation of the timer option, the investor specifies an expected investment horizon 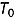 and a target volatility 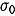 to define a variance budget

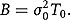

Let 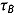 be the first time in the tenor of monitoring dates at which the discrete realized variance exceeds the variance budget *B*, namely

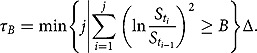

Let the current time be 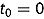, then the price of a finite-maturity discrete timer call option can be decomposed into two components, depending on whether the variance budget is consumed up before the mandatory maturity or not.

(4.1)
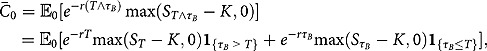

where *K* is the strike price.

The first presence of timer option in the literature dates back to the 1990s when Neuberger (1990) and Bick (1995) discuss the pricing and hedging of such an imaginary product. Since the official launch of timer option by Société Générale in 2008 (see Sawyer (2007)), research work that examines this exotic product from either an analytic or approximation perspective has been extensive (see Zeng, Kwok and Zheng (2015) and references therein). Voluminous as the literature is, most of the existing results are either achieved under the restrictive assumptions, such as perpetuity and continuous monitoring, or exhibit heavy computational burdens. For example, Li and Mercurio (2014) develop asymptotic formulas for pricing perpetual continuous timer options under general SVMs with volatility of variance as the perturbation parameter. As an extension, an approximation technique in Li and Mercurio (2015) is designed to work for pricing finite-maturity timer options under Heston-like models, but their analytic approximation approach still does not incorporate the discrete monitoring feature of the variance budget in actual timer option contracts. In addition, the condition that the effect of the volatility of variance is small is required for achieving sufficient accuracy in these two approximations. In fact, the direct investigation on the discrete timer option with finite maturity is relatively rare and is more mathematically challenging. Zeng, Kwok and Zheng (2015) construct the fast Hilbert transform algorithms for pricing finite-maturity discrete timer options under different types of stochastic volatility processes. Yet, their method is computationally expensive. In this subsection, we will discuss how to analytically price discrete timer options with finite maturity under the 3/2 model based on the newly derived partial transform of the triple density. Our proposed analytic pricing formula is almost exact and can be efficiently calculated by low-dimensional numerical integration.

As usual, we use the quadratic variation as a proxy of the discrete realized variance for the monitoring of the first hitting time to maintain the certain level of mathematical tractability. That is, we redefine 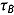 as follows:

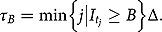

With this simplification, the price of a finite-maturity discrete timer call option can be conveniently computed by further decomposing it into a sequence of timerlets as follows:

(4.2)
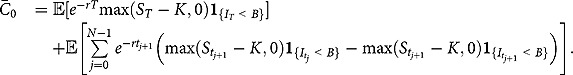

The key observation is that the event 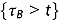 is equivalent to 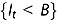. That explains the equivalence between the first term of (22) and (23). Other terms can be deduced similarly by noting that

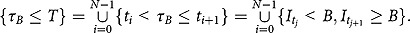

The above decomposition is first proposed by Lee (2013) and has been applied in Cui (2014).

The series of expectations in (4.2) can be easily evaluated by the standard transform method (e.g., Lee (2013)), if we can find the explicit expressions of the joint characteristic functions of 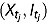 and 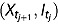. A direct application of Corollary 1 gives

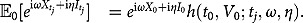

To compute the joint characteristic function of 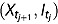, we follow the standard conditional expectation procedure and obtain

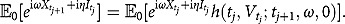

Apparently, the RHS of the above equation depends on the joint distribution of the triple 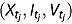 and cannot be computed by using Corollary 1 and Corollary 2 alone. In fact, the newly obtained partial transform of the triple density must be used here:

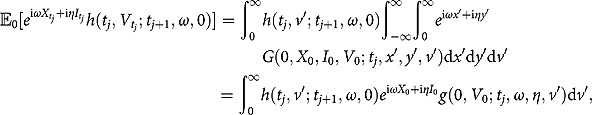

where the second equality follows from the observation that the inner double integral is exactly the definition of the partial transform (see (3.2) and (3.5)).

After finding the desired joint characteristic functions, we next calculate Fourier transforms of the payoff function. It turns out that the Fourier transform of 
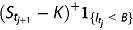
 and 
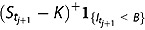
 admit the same analytic representation

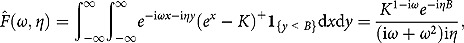

where 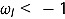 and 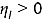. Finally, by Parseval’s theorem, the finite-maturity discrete timer option price can be derived as follows:

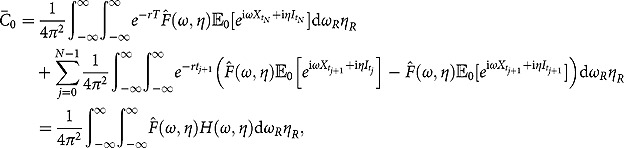

where

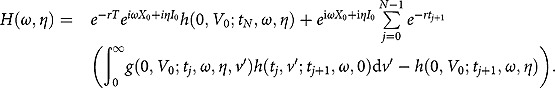

### 4.2. Discretely sampled weighted moment swaps

As a second example, we consider how the newly derived analytic results in Theorem 1 and its corollaries can be applied to price a rather general discretely sampled moment swap. Let 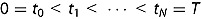 be 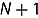 sampling dates and 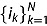 be a sequence of integers that take value from the index set 
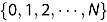
. We define the *k*th weight 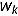 as a mapping: 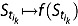 for some function *f*. Then, the floating leg of a weighted *m*th moment swap is defined by

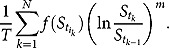

Immediately, we identify some special cases of discretely sampled weighted moment swapsin Table 1.

The pricing of the above weighted moment swap requires the computation of the fair strike price *K* which is set to be

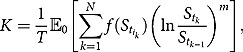

such that it costs zero for both parties to enter into the swap contract. Due to additivity of expectation, we have 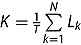, where

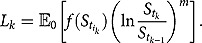

To compute 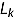, let us first consider the trivial case where 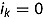 or 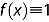, meaning that the weight is deterministic. As a result, we have

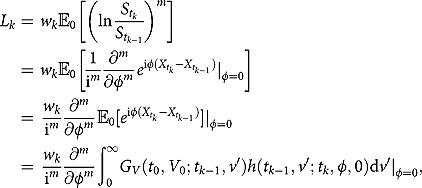

The above calculation applies to variance swap and skewness swap. Note that in the last second equality, the expectation is identified as the forward characteristic function first proposed by Hong (2004) and later used by Itkin and Carr (2010) to price discrete variance swaps.

Now, suppose 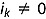 and *f* is also non-trivial and assume that *f* admits the generalized Fourier transform with respect to 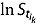 as follows:

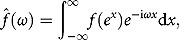

where the transform variable 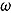 is a complex number and its imaginary part 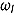 is fixed in a way such that 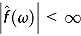. Then, we have

(4.3)
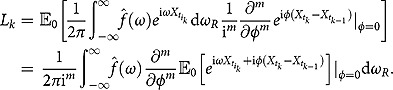

We remark that in (4.3), 
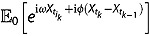
 is a model-specific term, whereas all other terms (including the differential operator) can be considered as product specific. This is a standard integral representation with the Fourier transform method, which holds for any pricing model.

In order to calculate the expectation in (4.3) under the 3/2 model, we first consider the scenario where 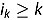. Using the tower rule and conditional expectation argument, we obtain

(4.4)
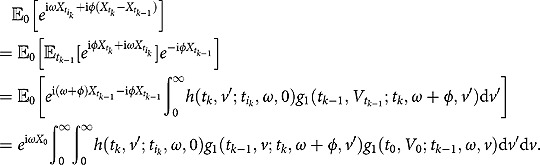

Specifically, when 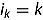, the double integral can be further simplified as a single integral. Since 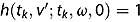 and 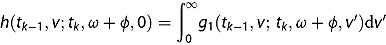, we have

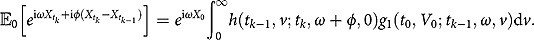

In a similar manner, we obtain the following formula for the case 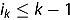:

(4.5)
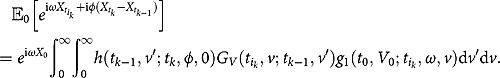

Further simplification is possible when 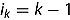. In that case, 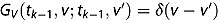, and hence we have

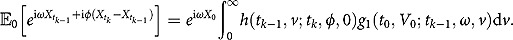

Observe that both (4.4) and (4.5) indicate that only one function in the integrand depends on the dummy variable 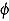. As a result, the differentiation with respect to 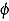 in (4.3) can be conveniently performed on that single function. The final formula for 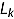 is obtained as a triple integral (a double integral in the two special cases) by plugging (4.4) or (4.5) into (4.3).

#### 4.2.1. Self-quantoed variance swaps

As an illustrative example, we consider the fair strike price of a self-quantoed variance swap. According to Table 1, its fair strike is computed by

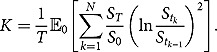

**Table 1.** Various weighted moment swaps.

Product			*m*
Variance swap	–		2
Gamma swap			2
Corridor variance swap	or		2
Self-quantoed swap			2
Skewness swap	–		3

Apparently, 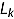 is now determined by (4.4). Moreover,

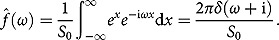

Consequently, the integration in (4.3) can be computed explicitly as follows:

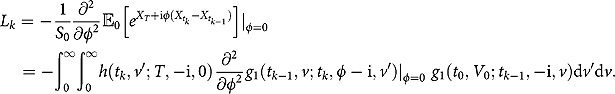

Therefore, we have

(4.6)
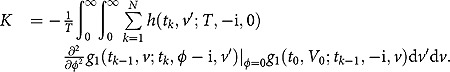

## 5. Numerical examples

In this section, we show some numerical examples that illustrate the performance of pricing formulas of the timer option and variance derivatives under the 3/2 SVM. We also examine the pricing behaviours with respect to varying model parameters.

Table 2 lists the default parameter values of the 3/2 model in our sample calculation. These parameter values, which have been calibrated to the S&P 500 option prices, are taken from Drimus (2012). All our numerical examples will use this set of parameter values unless specified differently.

**Table 2.** Parameter values in the 3/2 stochastic volatility model.

						*r*	*q*
.84	4.979	8.56	0.060025	−0.99	100	0.015	0

### 5.1. Finite-maturity discrete timer options

We set the strike price to be 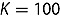, and the number of monitoring instants to be 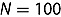. We also reset the value of correlation coefficient 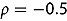 and initial variance 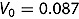 which are adopted in Zeng, Kwok and Zheng (2015) for comparison purposes. Considering that extensive analysis has already been done in Zeng, Kwok and Zheng (2015) and references therein, here we mainly explore the impact of variance budget on finite-maturity discrete timer option call prices over a wide range of maturities.

**Figure 1.**
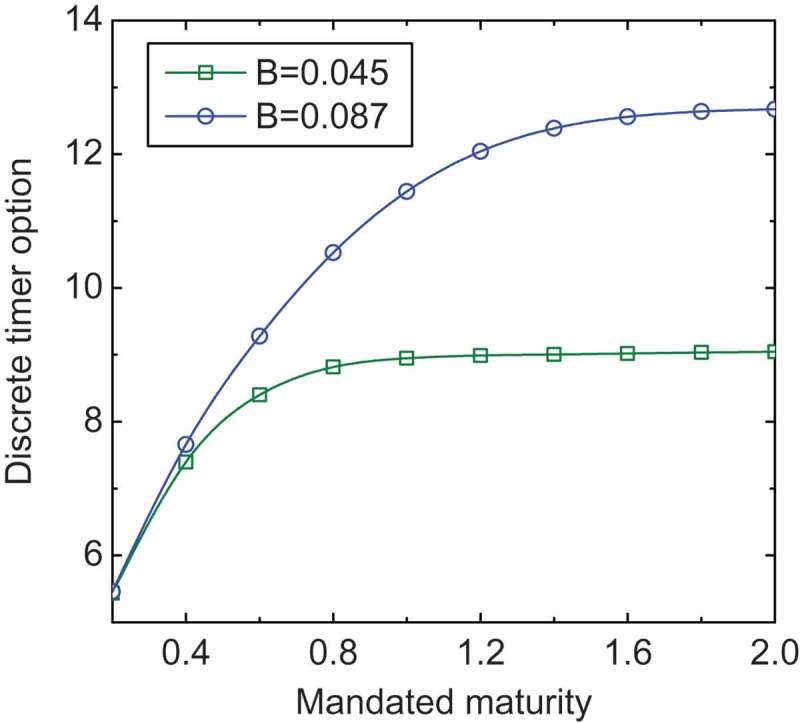
Plot of the finite-maturity discrete timer call option price versus mandated maturity given different values of variance budget.

Figure 1 shows that the finite-maturity discrete timer option price is an increasing function of both the maturity *T* and the variance budget *B*. Intuitively, an option is usually more expensive with a longer exercise time. The higher variance budget *B* or the longer the maturity *T* leads to the later arrival of the exercise, thus giving a more expensive timer option price. When *T* becomes sufficiently large, the finite-maturity discrete timer option value becomes almost insensitive to *T* and converges to that of the perpetual discrete timer call option from below. The finite-maturity discrete timer option value exhibits a more pronounced convergence given a lower *B*.

### 5.2. Self-quantoed variance swaps

**Figure 2.**
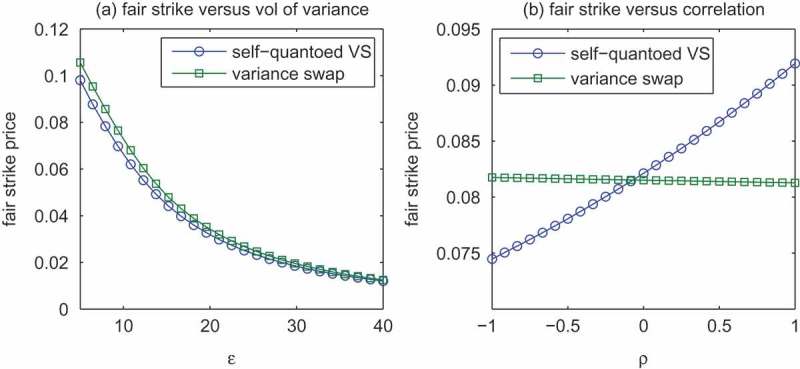
Comparison of the fair strike prices of a half-year daily sampled self-quantoed variance swap and a vanilla variance swap, with varying values of (a) vol of variance, (b) correlation coefficient.

We perform numerical analysis on the self-quantoed variance swap. Specifically, the fair strike prices of the self-quantoed variance swaps are compared with those of the variance swaps to exemplify the unique features of the former. In Figure 2, two plots of the fair strike price versus the correlation coefficient 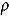 and volatility of variance 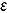 are given for half-year daily sampled swap contracts (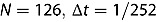).

In Figure 2(a), we vary the volatility of variance parameter while fixing other parameters at the values given by Table 2, to analyse the sensitivity of the fair strike prices of the self-quantoed variance swap and the vanilla variance swap to 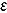. Within a realistic scope of 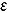, we observe large deviation in the fair strike prices of both swap products. Moreover, the fair strike prices are decreasing in 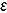. An intuitive explanation for this phenomenon can be found in Yuen, Zheng and Kwok (2015). By contrasting the self-quantoed variance swap to the variance swap, we see that the disparity between the two gets wider as 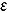 decreases. That’s because the protective feature of the self-quantoed variance swap begins to reinforce when the realized variance is getting large. A similar sensitivity analysis with respect to 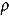 is performed in Figure 2(b). As expected, the self-quantoed variance swap is much more dependent on the correlation coefficient than the variance swap. When 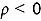, we have the renowned leverage effect and the self-quantoed variance swap is embedded with a crash protection for the seller. On the other hand, when 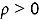, the soaring realized variance may be further exacerbated by the rising asset price, resulting in a larger risk exposure.

## 6. Conclusions

We explore the analytic tractability of the 3/2 SVM by investigating the joint distribution of the triple consisting of the log asset price, quadratic variation and instantaneous variance. We obtain the closed-form partial transform of the joint triple density under the 3/2 SVM with a time-dependent mean reversion parameter under two different formulations. In one approach, we establish the governing PDE of the partial transform which is then transformed to a Riccati system of ODEs and solved explicitly, while in the other one we relate the partial transform to the conditional characteristic function of the integrated variance and compute the latter one using a set of probabilistic tools, such as the change of measure technique. The newly derived partial transform serves as the underpinning of the transform-based methods for derivatives pricing under the 3/2 model and most extant characteristic functions or transforms can be viewed as marginal versions of the partial transform. As illustrative examples, the pricing formulas for the finite-maturity discrete timer options and self-quantoed variance swaps are expressed in terms of the partial transform.

## Appendix A. Proof of Theorem 1

It suffices to solve (3.4) which can be rewritten as

(A.1)

where . The terminal condition remains to be  To solve (A.1), we first change the variables  to  with  and . We then define  that satisfies

By expressing the partial derivatives of  in terms of the partial derivatives of *f*, we obtain the governing equation for *f* as follows:

subject to

Apparently, if we choose  such that

then the governing PDE of *f* becomes

(A.2)

where all the coefficients are affine in *u*. It follows that  can take two values

(A.3)

where

There are various ways of solving (A.2). We find the following approach that makes use of the Riccati system of ODEs the best. An alternative method is appended to the end of this proof.

### Riccati system of ODEs

We write the Laplace transform of *f* with respect to  as follows:

(A.4)

Then,  satisfies

(A.5)

subject to the terminal condition . By virtue of the affine structure of the PDE coefficients, (A.5) admits the following exponential affine solution:

(A.6)

where  and  are parameter functions determined by the following Riccati system of ODEs:

with boundary conditions  and . It can be found that (e.g., Zheng and Kwok (2014))

(A.7)

where

By noting that

we obtain

(A.8)

Plugging (A.7) and (A.8) into (A.6) yields

(A.9)

### Inverse Laplace transform

Finally, *f* can be retrieved by taking the inverse Laplace transform of  with respect to . For  for , the inverse Laplace transform of  is given by

(A.10)

where  is the modified Bessel function of the first kind. Closed-form expression of the inverse Laplace transform  exists if we can ensure that

(A.11)

where  means taking the real part. It will be shown later that this requires choosing the plus sign for  in (A.3) so that

Letting  and applying the inverse transform to , we have

Expressed in terms of *v* and , *g* is found to be

This completes the proof.

### An alternative method for solving (A.2)

Instead of performing the Laplace transform of *f* with respect to  and taking advantage of the Riccati system of ODEs, one may solve (A.2) using the method of characteristics. To proceed, we let , such that  satisfies the following PDE:

(A.12)

where . The terminal condition is given by

By applying the Laplace transform to  with respect to *u*:

the second-order PDE (A.12) is reduced to a first-order linear PDE:

(A.13)

subject to

**Lemma 2**. *The solution to (A.13) is given by*

(A.14)

where

*Proof*. Let  and . Then, (A.13) can be written as

(A.15)

with terminal condition: .

Notice that (A.15) is a first-order PDE and its characteristic equations are given by

If we define  and , then according to the characteristic equations, we have

As a result, the solution to (42) must have the following form:

where  is a function to be determined by the initial condition. Equivalently, we could rewrite the general solution in an explicit form of :

where  is a function to be determined by the initial condition.

Using the initial condition: , we obtain

As a result,  is given by

Therefore, the solution to (A.15) is given by

By noting that  and , we obtain (A.14).

Finally,  is obtained by taking inverse Laplace transform  which can be expressed explicitly, provided that

(A.16)

In this case, it requires choosing the minus sign for  in (A.3), so that

(A.17)

Using formula (A.10) and letting , we have

Expressed in terms of the original variables  and ,  is shown to have the same expression as (3.6).

### The choice for value of

When dealing with the inverse Laplace transform of  given by either (A.9) or (A.14), we need some technical condition which imposes constraints on the choice for value of . Here, we briefly discuss how different technical conditions lead to different choices for value of .

Denote . Since

we conclude that  under the usual branching line convention. Moreover,

implying that

In conclusion, we must have . Consequently, we have to choose  in order to meet (A.11) and  in order to satisfy (A.16).

## Appendix B. The 3/2 plus jumps model

In this section, we extend our results to the 3/2 plus jumps model, which is given by

(B.1)

where a jump component modelled by a compound Poisson process is appended to the asset price process of the original 3/2 model (1). Here,  is an independent Poisson process with constant intensity  and *J* stands for the random jump size which is assumed to be a normal random variable with mean  and variance . Moreover, the additional term in the drift  is the compensator for the jump component, where

With jumps in the asset price, the quadratic variation process is now given by , and the triple transition density *G* satisfies the following PIDE:

where  is the probability density of the jump size *J*. The partial transform defined by (3.2) admits a similar form given by

where *g* satisfies that following PDE:

The above PDE can be solved in the exactly same manner and the solution has the same expression as in (3.6) with a modified parameter  given by

## Appendix C. Proof of Theorem 2

To determine the new measure under which the calculation can be done conveniently, we need the following lemma. Let  be an open subset and  be a one-dimensional stationary time-inhomogeneous diffusion process satisfying

where  is a Brownian motion under the measure . Its infinitesimal generator is given by

We assume that  and  are bounded on the compact subsets of  for any fixed  and  in the open subset .

**Lemma 3**. *Assume*  *is twice differentiable and*  *for any* . *Define a new measure*  *by the Radon–Nikodym derivative*

(C.1)

*Suppose*  *does not explode and has unattainable boundaries under both Q and* , *then*  *is equivalent to Q and*

(C.2)

for any bounded measurable function , where  is the expectation taken under . Moreover, under the new measure , the dynamics of  is modified to be

(C.3)

where  is a -Brownian motion.

Lemma 3 is an extension of a related theorem established by Hurd and Kuznetsov (2008) from a general time-homogeneous diffusion process to a time-inhomogeneous diffusion process. Also, the function  can assume complex values. We give a sketch of the main ideas of the proof. Firstly, an application of the It formula for the process  yields

where  is a  local martingale and  is actually the stochastic exponential of . Since we assume that  does not explode and the boundary non-attainment conditions are satisfied under  and , following Cheridito, Filipović and Kimmel (2007), we can similarly derive that . Finally, the Girsanov measure change theorem implies (C.2) and (C.3).
Remark 7.
*The parameters in the functions of* ,  *and*  *should be also chosen to guarantee the boundary nonattaintment and nonexplosive properties of the process*  *under both*  *and* .

Equipped with Lemma 3, we are now able to obtain the conditional characteristic function of the integrated variance in closed form. Note that  and  can be evaluated in two different ways for any , where  is a CIR process specified by (3.11). On one hand, we consider the calculation of  based on Lemma 3. We first need to find a twice differentiable function  such that

(C.4)

for some constants . It can be easily verified that  is a solution. In fact, since  and , we have

Apparently, (C.4) holds if  and  is the solution to

which admits

Applying Lemma 3 to  with  and  yields

(C.5)

where  is the transition density of the CIR process  under the new measure . Moreover, the -dynamics of  is given by

(C.6)

where  is a -Brownian motion. According to Lemma 1, the transition density of  under  is given by

(C.7)

Notice that in order to make zero an unattainable boundary of  under , it follows that

(C.8)

On the other hand, we have the following alternative representation:

(C.9)

By equating (C.5) and (C.9), we obtain

Noticing that the above equation holds for any , we can then take the inverse Fourier transform with respect to  on both sides to obtain

(C.10)

Substituting (3.12), (C.7) and (C.8) into (C.10), we have

This immediately leads to (C.13).

## Appendix D. Proof of Corollary 1

Apparently, we have

Then, it suffices to compute

where . Using the equation

where , ,  and , we obtain

Here,

Note that the second equality follows from the identity:
